# Exposure to the Family Wellbeing program and associations with empowerment, health, family and cultural wellbeing outcomes for Aboriginal and Torres Strait Islander peoples: a cross-sectional analysis

**DOI:** 10.1186/s12889-023-16450-9

**Published:** 2023-08-18

**Authors:** Leonie Malezer Williamson, Leslie Baird, Komla Tsey, Yvonne Cadet-James, Mary Whiteside, Nadine Hunt, Raymond Lovett

**Affiliations:** 1 The National Centre for Epidemiology and Population Health, Australian National University, Canberra, ACT Australia; 2 Gurriny Yealamucka Health Service, Yarrabah, QLD Australia; 3 James Cook University, Smithfield, QLD Australia; 4 Apunipima Cape York Health, Cairns, QLD Australia

**Keywords:** Indigenous peoples, Aboriginal, Torres Strait Islander, Community empowerment, Health and wellbeing

## Abstract

**Background:**

Empowerment is an internationally recognised concept commonly incorporated in First Nations and in this instance Aboriginal and Torres Strait Islander health and wellbeing programs. The Family Wellbeing Program is an empowerment program developed in partnership with Aboriginal and Torres Strait Islander peoples that has been widely delivered to Aboriginal and Torres Strait Islander communities across Australia for close to 30 years. To date, there has been limited quantitative analysis of how this program is linked to health and empowerment outcomes.

**Methods:**

Cross sectional analysis of Mayi Kuwayu, the National Study of Aboriginal and Torres Strait Islander Wellbeing, baseline data (*n* = 9,843) recruited using multi-mode random sampling including mail out survey and in community convenience sampling. Logistic regression models were performed to calculate Prevalence Ratios (PRs) and 95% Confidence Intervals (CIs) to examine the association between personal control, life satisfaction, general health, family wellbeing and cultural wellbeing outcomes for Family Wellbeing participants (*n* = 718) versus non-participants (*n* = 9,125).

**Results:**

Compared with non-FWB participants, FWB participants are more likely to be female (67.1% versus 58.4%), be aged 35–54 (41.8% versus 32.0%) and live in a remote area (17.7% versus 10.4%) and have educational attainment at the Year 12 level or above (57.8% versus 53.2%). Family Wellbeing participation was associated with a 13% higher reporting of family functioning, a 74% higher reporting of cultural participation and a 21% in higher reporting of local decision making in the local community compared to non-FWB participants. There were significant associations between FWB exposure compared to non-FWB exposure including reporting lower levels of health risk factors including quitting alcohol (26.4% versus 20.4%), regular exercise (67.7% versus 66.3%), quitting smoking (33.4% versus 31.9%). and e. FWB participants who had experienced both prison and youth detention were nearly double that of Non-FWB (3.5% versus 1.4%) and more reported being removed from their families as children (Stolen) (7.0% versus 4.1% Non-FWB).

**Conclusion:**

There are significant associations between Family Wellbeing exposure and organisation and community level empowerment outcomes, but only for some individual level empowerment outcomes. There is a lower reporting health risk factors including increased physical exercise, reduced alcohol use and smoking; and educational attainment among FWB participants compared to non-FWB participants. The results suggest individual, community and organisational empowerment needs to be explored further with more robust study designs that can attribute causality and direction of association.

**Supplementary Information:**

The online version contains supplementary material available at 10.1186/s12889-023-16450-9.

## Introduction

Empowerment theory has become a common concept in public health since the World Health Organisations’ 1986 Ottawa Charter recognised ‘the process of enabling people to increase control over, and to improve, their health’ as vital to creating global health equity [1]. Central to empowerment theory is the recognition of power or powerlessness as a core social determinant, thus there is concern with not only the outcomes of good health and wellbeing, but also the processes of control over the determinants by which good health is achieved [2, 3]. Observation and analysis of these processes and outcomes occurs across three broad levels, personal or psychological empowerment, organisational empowerment and community empowerment [3–5]. While individual behaviours and attributes relating to agency and self-efficacy are commonly associated with empowerment, the theory is much broader and also concerned with the participatory action processes that galvanise individuals towards community action, and the structures that may support or impede them [2–6].

Empowerment is complimentary to Self-determination which comes from an international rights framework (UNDRIP, 2007) and is commonly advocated for by Indigenous communities worldwide. Self-determination is a long standing legal and policy concept advocated by Aboriginal and Torres Strait Islander peoples since the early 1970’s and is a dynamic right to make decisions and to control their implementation. While there are differences, empowerment can be thought of in this instance as the processes of realising self-determination.

Wallerstein’s work on empowerment as a health enhancing strategy asserts that understanding the role of social protective factors is key to developing effective theory of change models that can reduce ill health brought about by social inequity [3]. This approach challenged what was seen as a reductionist approach to empowerment in public policy and media where emphasis was placed on personal agency and self-efficacy, expressed as a lack of personal responsibility or drive, instead of highlighting broader social problems. Wallerstein argued for the term ‘community empowerment’ emphasising the participatory processes and social justice approach underlying the concept (p.74, [3]).

Community empowerment theorists pointed out that consideration must be made to variations in protective factors by population, geography and time [3, 7]. They also point to the dynamic nature of the processes and by which they relate [5] and explain that empowerment remains a model still largely reliant on the social-determinants to explain inequity. There is however a growing body of work on the limitations of social determinants to explain health inequity for Indigenous Peoples, that calls for a greater emphasis on culture in the understanding of health risk and protective factors [8–12]. Therefore, gaps remain in the adaption of community empowerment models.

The Family Wellbeing program (FWB) was developed in 1993, conceived in consultation with a group of survivors of the stolen generations. The stolen generation were and are Aboriginal people forcibly removed from their families and were required by the state to undertake domestic or agricultural labour training, then be in the employ of non-Indigenous families from the early 1900’s through to the end of the 17,970’s. The Family Wellbeing program it is a group learning program that focuses on five stages of personal, family and community empowerment. Initiated as an employment and training program it has been adapted and delivered for a range of community identified needs and audiences; including suicide prevention, family violence, inmate education and parenting support [13]. Commonly delivered as a train the trainer model whereby organisation staff participate in the course and are then able to apply the skills and knowledge to work settings and interactions with service users. Family Wellbeing includes a short course for participants on understanding and meeting basic human needs like shelter, exercise, food, sleep. It then also discusses concepts including identity, sexual expression, respect for self and others, life-long learning and connecting to something greater than the individual. Further, it offers problem-solving skills that help participants build strengths in managing relationships, conflicts, addiction and violence. The short course is flexible in delivery and can run over several weeks. It is often delivered in flexible modes to suit community needs and each module has 30 h of group learning (see Table 1).

**Table 1:** The five stages of Family Wellbeing Empowerment Programme (18)

FWB stage	
Stage 1: Human qualities	Introduction to core FWB concepts including human qualities, basic physical, mental, emotional and spiritual needs of life, exploring traditions and values, analytic tools for self-reflection and skills for self-care and providing counselling and support to others
Stage 2: The process of change	How change affects people, how it can be experienced as an opportunity to grow and develop by recognising and building on inner qualities and strengths, and the importance of framing difficulties as challenges for which there are always solutions rather than as problems
Stage 3: Changing the patterns	Applying FWB approach to issues of family violence and abuse and other social dysfunction
Stage 4: Opening the Heart	Reinforcing FWB messages of self-development, healing and healthy relationships
Stage 5: FWB facilitation	Practise-based training for FWB facilitation

The FWB has been delivered in 60 known sites across Australia with more than 5,400 participants [13, 14].

Existing studies and evaluations of the FWB have analysed the applicability of empowerment used in the program to Aboriginal and Torres Strait Islander contexts [15–17], the demand driven transfer of the program across sites and services [13] personal empowerment outcomes [17, 18], the impact of research attached to the program and its application as a suicide prevention intervention [19]. Findings of these studies have included improvements in personal empowerment outcomes amongst FWB participants [17], the program as a mechanism for local action and organisation (community empowerment) [19] and the possible limitations of empowerment frameworks to Aboriginal and Torres Strait Islander world views that emphasise cultural and spiritual connections [15]. These studies have been qualitative and limited quantitative studies of FWB participation on health and wellbeing outcomes have occurred [16, 17].

This analysis examined the relationship between exposure to the FWB on health, wellbeing, cultural and empowerment outcomes.

## Materials and methods

Mayi Kuwayu: The National Study of Aboriginal and Torres Strait Islander Wellbeing (the Mayi Kuwayu Study) is a longitudinal study that has been designed to quantify the links between social and cultural determinants, such as cultural expression, community decision making and family connection, to health and wellbeing [8, 9]. It is the largest prospective cohort study of Aboriginal and Torres Strait Islander peoples in Australia. The study is open to participation by Aboriginal and Torres Strait Islander adults aged 16 and over and commenced recruitment in 2018. Participants were recruited via mixed methods, including through the Medicare, through in-community recruitment or through online questionnaire [20]. All Mayi Kuwayu Study data included in the current analysis were based on self-reported responses, with the exception of remoteness, which was derived from postcode.

This cross-sectional analysis is conducted using the third release (R.3) of the Mayi Kuwayu baseline data (*N* = 9,843), and includes all responses received between 2018-December 2020. FWB participants are identified by a question asking ‘Have you ever participated in: the Family Wellbeing Program?’, non-FWB were any survey participant who did not select the “Family Wellbeing Program” option. No information was collected on the date of participation in the program.

The measures of culture and wellbeing have been developed through extensive research led by Aboriginal and Torres Strait Islander people including extensive partnerships, consultation and field testing with communities [8, 9, 21, 22].

### Governance and ethics

The study is overseen by an Aboriginal and Torres Strait Islander governance group consisting of peak Aboriginal and Torres Strait Islander health organisations. In addition, all data use is assessed according to Indigenous Data Sovereignty principles and approved by an independent Indigenous Data Governance committee, known as the Mayi Kuwayu Data Governance Committee (Approval Reference No. D210511). The research was conducted with ethics approvals from relevant Aboriginal and Torres Strait Islander organisations, and from national, state and territory Human Research Ethics Committees (HRECs).

### Variables

#### Exposure variable (FWB participation)

Family Wellbeing participants are identified through Mayi Kuwayu question. *‘Have you ever participated in: Family Wellbeing Program’*.

#### Outcome variables

Outcome variables used in the study were selected based on analysis of the literature, including previous evaluations of the program [16, 17, 23] and feedback from program providers. They are designed to provide indicators across personal, organisational and community empowerment, and health outcomes (see Appendix 1).

#### Personal empowerment

Personal empowerment was measured with responses to the question, “how much are you in control of your life?”’, with response options “A lot”, “A fair bit”, “A little bit” and “Not at all”. These were then dichotomised as a (0) not at all and (1) little—a lot. Life satisfaction was measured according to responses to the question, “How satisfied are you with your life?”, with response options “a lot”, “a fair bit”, “a little bit” and “not at all”. These were then categorized as a (0) not at all and (1) little to a lot. General health was measured according to the question “How would you rate your general health?”; response options were “poor”, “fair”, “good”, “very good” or “excellent”. Responses were categorised as (0) poor-fair and (1) good–excellent.

Family functioning was measured according to a composite score of responses to a set of nine questions asking “In my family…”, “We get together and cope in the hard times”, “we celebrate special days/ events”, “we talk with each other about the things that matter”, “we are always there for each other”, “we manage money well”, “we have common interests”, “people are accepted for who they are”, “we have good support from mob”, “we have family knowledge and traditions that we pass on to our children”. With response options “not at all” (1), “a little bit” (2), “a fair bit” (3) to “a lot” (4), and “unsure” (0). Responses were summed (range: 0–36), and participants were categorized as having (0) low- moderate family functionality (score 0–29) or (1) high family functionality (score 30–36). Responses to the nine questions were summed for participants with complete data only; participants missing responses to any of the questions were coded as missing. All outcome measures above were coded as binary for use in the regression analysis.

#### Organisational empowerment

For organisational empowerment a composite score of the cultural knowledge and practice subset was created, that includes questions relating to contribution to community and participation in community-based events. The score was made up of responses to ten questions, summed together to generate a total cultural wellbeing score ranging from zero to 40. The questions asked “How much time do you spend…” 1. “With someone who has cultural knowledge (elder or knowledge holder)?” 2. “Learning and using knowledge from Aboriginal/Torres Strait Islander Law (or Lore)?” 3. “On country?” 4. “Getting or eating bush tucker (includes traditional foods and fishing)?” 5. “Learning culture, kinship and respect?” 6. “Making art, music or painting?” 7. “Passing on cultural knowledge?” 8. “Participating in social events related to Aboriginal/Torres Strait Islander people (such as NAIDOC week, Sorry Day events, cultural festivals, corrobboree, marches or rallies)?” 9. “Contributing to your community (such as participating in community meetings, organising events, volunteering, healing, being on committees or boards)?” 10. “Receiving Aboriginal/Torres Strait Islander healing methods (such as traditional healers, bush medicine)?”. With response options "Not at all" (1), "A little bit" (2), "A fair bit" (3) and "A lot" (4). Responses for the 10 questions were summed together to generate a total cultural wellbeing score ranging from zero to 40; participants missing any data had their score coded as missing. A binary category was created of (0) low cultural wellbeing (scores of less than or equal to 15) and (1) high cultural wellbeing (scores between 15 and 40).

#### Community empowerment

Decision making was measured by the question “in the Aboriginal and/ or Torres Strait Islander community where I live now local Aboriginal and Torres Strait Islander people make community decisions” with response options “Not at all”, “A little bit”, “A fair bit”, “A lot” and “Unsure”. Responses were coded and categorised to (0) not at all (Not at all) and (1) A little – a lot; unsure was coded as missing.

##### Health seeking and health risk factors and wellbeing variable

The presence of a drug and/ or alcohol problem was measured by response to the question that asked, “has a doctor ever told you that you have…drug or alcohol problem” with responses “no” and “yes”. Participants were also asked “do you drink alcohol?” with responses categorised as “current drinker”, “ex-drinker” and “never drinker”. Current smoker was measured with response to question “Do you smoke?”, with response categories created for “current smoker”, ex-smoker” and “never smoker”. Current smokers were also asked “do you want to quit smoking?” with five response options that were categorised as “not at all or unsure” and “a little to a lot”. For exercise in the past week where participants were asked to select the days that they did 30 or more minutes of exercise (Monday – Sunday) the responses (1 for each day selected) were summed into three categories “none”, “1–2 days” and “3 or more”.

#### Demographic variables

Eight demographic variables were used in the study including gender, age, location, and education. Following consultation with FWB providers we added measures for incarceration, low income, being removed as a child (Stolen^1^) and household overcrowding, as these were priority groups for the program over the last 30 years. Gender was coded according to response of “male” or “female”, with “other” coded as missing due to the small number of responses. Age was recoded into three age categories “16 to 34 years”, “35 to 54 years”, and “55 + years”. Education was recoded into two categories “Year 10 or below” (no school, primary school, some high school, and year 10) and “Year 12 or above” (Year 12, certificate or diploma and university). For the location variable the categories were collapsed to, “regional” (inner regional and outer regional), “remote” (remote and very remote) and “major cities” (major cities). Incarceration was measured from participant response to the question “Have you ever been in prison or youth detention?”, with categories created for “no prison or youth detention”, “prison”, “youth detention”, "youth detention and prison"; participants in the “prison and youth detention category” were omitted from the single response “prison” and “youth detention” categories. Family financial status was measured from response to the question of “which words best describe your family's money situation?” with the six response options collapsed to the categories of “we have enough” ( we have a lot of savings, we have some savings and we have just enough to get us to the next payday) and “we don’t have enough” (we run out of money before payday, we are spending more than we get); the response of unsure was coded as missing. Participants were asked ‘Is where you live crowded?’ with five response options collapsed to “Not at all” and “a little to a lot”, the response not relevant was coded as missing. Stolen generation was measured with response to the question “were any of these people Stolen? With response options of “I was Stolen” or “I was not Stolen”.

### Statistical analysis

A descriptive analysis is provided of the socio-demographic variables (Table 2), health risk factors and behaviours (Table 3) and empowerment outcome (Table 4) presented as percentage and number (%, *n*) overall and by FWB exposure. A Chi-square statistic was calculated to test for significant differences between FWB exposure and the categories of each demographic variable (Table 5).

**Table 2:** Demographic characteristics of Mayi Kuwayu Study participants overall and by FWB/non-FWB participation

	**Total (*N* = 9,843)**	**FWB participant (*n* = 718)**	**Non-FWB participant (*n* = 9,125)**
		% (*n*)	
**Gender^**			
Male	37.9 (3,729)	30.6 (220)	38.5 (3,509)
Female	59.5 (5,858)	67.1 (482)	58.9 (5,376)
Missing	2.6 (256)	2.2 (16)	2.6 (240)
**Age group (years)^**			
16–34	23.1 (2,270)	16.2 (116)	23.6 (2,154)
35–54	32.7 (3,222)	41.8 (300)	32.0 (2,922)
55 +	40.9 (4,024)	39.4 (283)	41.0 (3,741)
Missing	3.3 (327)	2.7 (19)	3.4 (308)
**Location^**			
Major City	41.1 (4,048)	32.0 (230)	41.8 (3,818)
Regional	47.6 (4,681)	49.9 (358)	47.4 (4,323)
Remote	10.9 (1,072)	17.7 (127)	10.4 (945)
Missing	0.4 (42)	0.4 (< 5)	0.4 (39)
**Education^**			
≤ Yr 10	44.8 (4,408)	40.0 (287)	45.2 (4,121)
≥ Yr12	53.2 (5,234)	57.8 (415)	52.8 (4,819)
Missing	2.0 (201)	2.2 (16)	2.0 (185)
**Family financial status**			
We have enough	73.6 (7,243)	72.6 (521)	73.7 (6,722)
We don't have enough	15.9 (1,561)	17.1 (123)	15.8 (1,438)
Missing	10.6 (1,039)	10.3 (74)	10.6 (965)
**Is where you live crowded^**			
Not at all	74.5 (7,329)	69.1 (496)	74.9 (6,833)
A little to a lot	16.7 (1,645)	21.6 (155)	16.3 (1,490)
Missing	8.8 (869)	9.3 (67)	8.8 (802)
**Incarceration^**			
No	88.2 (8,685)	83.8 (602)	88.6 (8,083)
Prison	8.3 (820)	10.7 (77)	8.1 (743)
Youth Detention	1.8 (181)	2.0 (14)	1.8 (167)
Prison & Youth Detention	1.6 (157)	3.5 (25)	1.5 (132)
**Stolen^**			
Not Stolen	95.7 (9,423)	93.0 (668)	96.0 (8,755)
Stolen	4.3 (420)	7.0 (50)	4.1 (370)

^sig < 0.05

**Table 3:** Health behaviours and risk factors in the Mayi Kuwayu by FWB/non-FWB participation

	**Total (*N* = 9,843)**	**FWB participant (*n* = 718)**	**Non-FWB participant (*n* = 9,125)**
		% (*n*)	
**General health**			
Good–Excellent	66.6 (6,551)	63.4 (455)	66.8 (6,096)
Poor—Fair	30.9 (3,038)	33.8 (243)	30.6 (2,795)
Missing	2.6 (254)	2.8 (20)	2.6 (234)
**Diagnosed drug/ Alcohol problem**			
No	93.7 (9,220)	92.1 (661)	93.8 (8,559)
Yes	6.3 (623)	7.9 (57)	6.2 (566)
**Do you drink alcohol^**			
Current drinker	57.9 (5,694)	53.2 (382)	58.2 (5,312)
Ex-drinker	20.8 (2,049)	26.2 (188)	20.4 (1,861)
Never a drinker	18.2 (1,793)	18.4 (132)	18.2 (1,661)
Missing	3.1 (307)	2.2 (16)	3.2 (291)
**Smoking status^**			
Current Smoker	25.9 (2,549)	29.4 (211)	25.6 (2,338)
Ex-Smoker	32.0 (3,151)	33.4 (240)	31.9 (2,911)
Never a Smoker	39.4 (3,879)	35.1 (252)	39.8 (3,627)
Missing	2.7 (264)	2.1 (15)	2.7 (249)
Quit intentions*			
Not at all or unsure	23.1 (601)	20.2 (43)	23.4 (558)
A little to a lot	76.9 (1,998)	79.8 (170)	76.6 (1,828)
**Exercise in the past week^**			
None	21.8 (2,141)	18.3 (131)	22.0 (2,010)
1–2 Days	11.8 (1,163)	14.1 (101)	11.6 (1,062)
3 or more days	66.4 (6,539)	67.7 (486)	66.3 (6,053)

^sig < 0.05

^*^n = *2599, FWB* = *213, non FWB* = *2386*

**Table 4:** Empowerment, health, and wellbeing outcomes overall and by FWB/Non-FWB participation

	**Total (*N* = 9,843)**	**FWB participant (*n* = 718)**	**Non-FWB participant (*n* = 9,125)**
		% (*n*)	
**Personal Empowerment**			
**Life control**			
A little to a lot	94.0 (9252)	93.7 (673)	94.0 (8,579)
Not at all	2.5 (249)	2.8 (20)	2.5 (229)
Missing	3.5 (342)	3.5 (25)	3.5 (317)
**Life satisfaction**			
A little to a lot	91.6 (9,012)	91.6 (658)	91.6 (8,354)
Not at all	5.1 (505)	5.0 (36)	5.1 (469)
Missing	3.3 (326)	3.3 (24)	3.3 (302)
**Family functioning^**			
High-very high family functioning	34.8 (3,426)	45.7 (328)	34.0 (3,098)
Low-mod family functioning	40.8 (4,014)	43 (309)	40.6 (3,705)
Missing	24.4 (2,403)	11.3 (81)	25.5 (2,322)
**Organisation empowerment**			
**Cultural Wellbeing^**			
High-very high	41.2 (4,058)	70.9 (509)	38.9 (3,549)
Low-moderate	45.1 (4,443)	19.1 (137)	47.2 (4,306)
Missing	13.6 (1,342)	10.0 (72)	13.9 (1,270)
**Community empowerment**			
**Decision making^**			
A little to a lot	47.9 (4,710)	74.5 (535)	45.8 (4,175)
Not at all	16.8 (1,658)	10.7 (77)	17.3 (1,581)
Missing & unsure	35.3 (3,475)	14.8 (106)	36.9 (3,369)

^sig < 0.05

**Table 5:** Relationship between FWB empowerment, health and wellbeing outcomes

	**Crude PR (95%CI)**	**PR Adjusted for Gender (95%CI)**	**PR Adjusted for Age (95%CI)**	**PR Adjusted for Location (95%CI)**	**PR Adjusted for ex-Drinker (95%CI)**
**Life control (a little—a lot)**					
Non-FWB	1 (ref)	1 (ref)	1 (ref)	1 (ref)	1(ref)
FWB Participant	1 (1.00–1.00)	1 (1.00–1.00)	1 (0.99–1.00)	1 (1.00–1.00)	1 (0.99–1.00)
**Life satisfaction (a little—a lot)**					
Non-FWB	1 (ref)	1 (ref)	1 (ref)	1 (ref)	1(ref)
FWB Participant	1 (0.98–1.02)	1 ((0.98–1.02)	1 (0.98–1.02)	1 (0.99–1.02)	1 (0.98–1.02)
**Community decision making (a little—a lot)**					
Non-FWB	1 (ref)	1 (ref)	1 (ref)	1 (ref)	1(ref)
FWB Participant	1.21 (1.16–1.25)	1.20 (1.15–1.24)	1.20 (1.16–1.24)	1.15 (1.12–1.18)	1.20 (1.16–1.24)
**General health (good–excellent)**					
Non-FWB	1 (ref)	1 (ref)	1 (ref)	1 (ref)	1(ref)
FWB Participant	0.95 (0.90–1.01)	0.95 (0.89–1.00)	0.97 (0.91–1.02)	0.95 (0.89–1.00)	0.95 (0.90–1.00)
**Family functioning (high-very high)**					
Non-FWB	1 (ref)	1 (ref)	1 (ref)	1 (ref)	1(ref)
FWB Participant	1.13 (1.04–1.22)	1.12 (1.04–1.21)	1.12 (1.04–1.22)	1.11 (1.02–1.20)	1.12 (1.04–1.22)
Cultural Wellbeing (high-very high)*					
Non-FWB	1 (ref)	1 (ref)	1 (ref)	1 (ref)	1(ref)
FWB Participant	1.74 (1.66–1.83)	1.74 (1.66–1.82)	1.72 (1.64–1.81)		1.13 (1.04–1.22)

^*^missing adjustment model for location

Logistic regression was performed to examine the association between FWB participation and each outcome. Prevalence Ratios (PRs) and 95% Confidence Intervals (CIs) are presented for each exposure/outcome association. Models are presented unadjusted and adjusted accounting for gender, age, location and being an ex-drinker as these were conceptually thought to be linked to both exposure and outcome variables. A sensitivity analysis was conducted to address possible contamination within the analysis with Family Wellbeing Services [24] run by the Queensland Government. Analysis that included, and then excluded, all Queensland Family Wellbeing participants was run. Participants with missing data on each outcome of interest were excluded from the study. Analysis was conducted using Stata 16 [25].

## Results

### Participant characteristics

Of the total study population (9,843), 718 indicated participating in the FWB program and 9,125 did not indicate participation in FWB. Around 6 in 10 (59.5%) were female and the predominant age category was 55 + (40.9%). Close to half of the study respondents lived in a regional area (47.6%), with 41.1% residing in a major city and 10.9% residing in a remote area.

There were a number of significant differences (*p* < 0.05) observed between FWB and non-FWB participants including by gender with around two-thirds (67.1%) of FWB participants being female (versus 58.4% Non-FWB), being in the 35-54 yr age category (FWB 41.8%, versus 32.0% Non-FWB participants). Regional areas were the most common area of residence for both FWB (49.4%) and Non-FWB (47.4%), however more of the FWB group resided in remote areas (17.7%) compared to 10.4% of non-FWB participants. Less than one third of FWB participants (32.0%) lived in a major city (versus 41.8% non-FWB).

Family wellbeing participants reported a significantly higher level of educational attainment of (Year 12 or above education FWB 57.8% versus 52.8% Non-FWB). There was no difference in family financial status between the two groups. A significantly higher level of overcrowding was reported by FWB participants (21.6% versus 16.3% Non-FWB).

There were significant differences in reporting of incarceration and stolen generations between FWB and non-FWB participants with 7% of FWB participants reporting being stolen or removed as children compared to 4% of Non-FWB participants. Most FWB participants (83.3%) reported no experience of incarceration (versus 88.6% non-FWB). Overall FWB participants reported a significantly higher level of both prison and youth detention (3.5% versus 1.4% non-FWB).

### Health outcomes and risk factors

Overall, 6.3% of Mayi Kuwayu participants reported a diagnosis of a drug or alcohol problem by a doctor (Table 3). Close to 6 in 10 participants (57.8%) reported being a current drinker, while nearly 1 in 5 (18.2%) reported never consuming alcohol. Just over one quarter of participants were current smokers (25.9%) and of those, 76.9% wanted to quit. Two-thirds of participants (66.4%) reported exercising for at least 30 min three or more days per week while 21.8% had done no exercise in the past week.

There were no significant differences in reporting of general health or diagnosis of a drug or alcohol problem between FWB participants and non-participants. Significant differences between the two groups were observed for drinking status, smoking status, desire to quit smoking and exercise participation in the last week. Over a quarter (26.4%) of FWB participants had stopped drinking (versus 20.4% Non-FWB) and around a third 33.4% had quit smoking (31.9% Non-FWB). Of current smokers 79.8% in the FWB participant group reported wanting to quit smoking, compared to 76.6% of Non-FWB smokers. The majority of FWB participants (67.7%) had undertaken 3 or more days of exercise in the past week, (versus 66.3% non-FWB), less than one-fifth (18.3%) of the exposed group reported having done no exercise in the past week to 22% of the non-FWB.

### Empowerment (personal, organisational and community), health and wellbeing

Against the personal empowerment outcomes, 94.2% of all participants reported a little to a lot of personal control, similarly 91.6% reported a little to a lot of life satisfaction (91.6%). Against the organisational empowerment outcome 41.2% of participants reported high levels cultural wellbeing. Around half of survey participants (47.9%) felt that local people made decisions in the community that they lived.

The prevalence of high personal control and life satisfaction was similar across FWB and Non-FWB participants. There were significant differences on family and community empowerment including almost half of FWB participants (45.7%) reporting high-very high outcomes on the family functionality scale (versus 34% Non-FWB) and 70.9% reporting moderate-high cultural wellbeing (versus 38.9% Non-FWB). Almost three-quarters (74.5%) of FWB participants reported that local Aboriginal and/ or Torres Strait Islander people made decisions in their community, compared to less than half of the Non-FWB group (45.8%).

### Associations between FWB, empowerment, health, and wellbeing

There were no significant differences in life control PR 1.00 (CI. 1.00–1.00), life satisfaction PR 1.00 (0.98–1.02) or general health 0.95 (0.90–1.02) between FWB participants and non-Participants. There was a significant 13% increase in reporting high levels of family wellbeing PR 1.13 (CI 1.04–1.22); a 74% increase in reporting high levels of cultural wellbeing PR 1.74 (CI 1.66–1.83); and a 21% increase in reporting high levels of local decision making by Aboriginal and Torres Strait Islander people PR 1.21 (CI 1.16–1.25). After adjustment for gender, age, location and being an ex-drinker there was no difference in all but one exposure outcome relationship. Being a former drinker did reduce the prevalence ratio of cultural wellbeing to a 13% increase from a 74% increase in the unadjusted model.

## Discussion

In this cohort of Aboriginal and Torres Strait Islander adults we observed significant associations between FWB participation and higher reporting of some outcomes including cultural wellbeing, community empowerment and for high-very high family functioning (13%). We did not observe a significant association between FWB participation, personal empowerment and general health. We did observe significant associations between FWB participation and increased prevalence of addressing health risk factors including quitting smoking, increased physical inactivity and quitting alcohol. We also observed a significant increase in year 12 or higher educational attainment amongst FWB participants compared to Non-FWB (57.8% v 52.8%) participants.

These findings are in line with previous qualitative studies where participants reported wanting to connect with their spirituality. This accords with previous qualitative FWB studies where participants felt that colonisation had undermined Indigenous culture and spirituality [18]. This may be one reason that explains the higher reporting of cultural wellbeing in this study where participants felt able to reconnect with culture, spirituality and family. The results of this study in individual health and risk factors also accord with previous FWB qualitative analyses where participants were able to indicate that they were better able to cope with the daily challenges of life, reduce stress which resulted in healthier lifestyles such as drinking less and increasing physical activity [18, 26]. An increased contribution in the community and family functioning where FWB participants lived has also been previously identified as a positive outcome from the program as was the case in our analysis. The existing evidence showed that family and community participation came about through FWB providing the skills to facilitate new attitudes and provide skills to engage in respectful relationships at the family and community level [18].

The higher prevalence of participation in the FWB program by people who live remote areas, have been Stolen and experienced incarceration (youth detention, jail or both), accords with a history of program delivery for stolen generations and those experiencing incarceration and supports the flexibility of the program to address local needs identified by communities, including removal and incarceration [13]. The outcomes suggest a pathway from increased organisational and community empowerment indicators to positive changes in health risk factors may be occurring. While there was no significant association between Participants exposed to FWB program do have a different demographic profile to those unexposed, which may affect generalisability of findings to other settings and therefore careful consideration of the mechanisms of implementation are required. However, internal comparisons are generalizable. FWB program exposure and general health, the significantly higher reporting of exercise and education, may manifest in health gains in the longer term. This is in line with community empowerment theory that describes empowerment interventions as having both a direct and indirect effect on health and wellbeing outcomes [3]. What the results may be suggesting is that engagement with, and contribution to, community and cultural life is linked to the adoption of healthy habits and therefore may be an intermediary to improved health outcomes [3, 18].

It is unclear why there is no significant association between FWB program exposure and life control and life satisfaction in this study, given both the strong focus in the program on individual healing and wellbeing, and the associated improvements in the outcome measures of family functioning, cultural wellbeing and community decision making. However, the findings are still in line with definitions of empowerment processes as a dynamic continuum [3, 5]; whereby individual agency may drive participation in community organisation, so too can participation and community connection be a driver of improved psychological wellbeing and health.

A limitation of this study is that it is cross-sectional and therefore no causality can be attributed and we are also unable to assess the direction of association. Another limitation is the high scores in life satisfaction and control across both the exposed and non-exposed group, contributing to a ‘ceiling effect’ which limits variation in outcomes between the two groups. To overcome these limitations, exploration of how these outcomes change over time for participants using more robust study designs would be desirable. The possible confusion of the program with other similarly named programs or services was also raised as a possible weakness of the study, as stated previously this was a particular concern in Queensland, however the sensitivity analysis identified no significant difference and therefore all Queensland participants were included in the final analysis. We note that mandating of the program may have occurred in some areas but we were unable to determine if participants were mandated, which may impact on the results. There is also a question of cultural nuance that requires adaption to the levels of analysis, or domains within them, to be more in line with Aboriginal and Torres Strait Islander cultural understandings of self. This would be in line with earlier qualitative evaluations of the Family Wellbeing program that found participants placed particular emphasis on relational and spiritual traits as attributes of empowerment [15, 18].

Missing from the analysis is information on structural change, or evidence of how an empowerment intervention such as the Family Wellbeing program can influence changed conditions, different policies or impact experiences of racism. For example, the ability of participants to improve their housing conditions in remote areas where housing infrastructure is limited and over-crowding more common [27]. There are limitations to the difference individual and, or, collective action can make to health and wellbeing without changes to the structural inequality that creates the need for empowerment interventions in the first place: not recognising this risks attributing responsibility for outcomes solely at the agency of the individual or community [3, 28, 29]. A larger more mixed method study design would be required to capture if and how such changes occur, and the processes by which community advocacy plays a part.

## Conclusion

The FWB program was significantly associated with higher reporting of cultural wellbeing, family functioning and local decision-making, reporting included reduction of risk factors, such as drinking and smoking, implies a model whereby both immediate and intermediate effects when compared to non-FWB participants. These positive associations are encouraging, particularly in combination with past consistent qualitative evidence of program benefits. A fuller evaluation of this program is warranted, using more robust study designs to provide a prospective quantification of the benefits of participation.

### Supplementary Information

**Additional file 1**: **Appendix 1. **Framework for empowerment evaluation outcomes.

## Data Availability

The datasets analysed during the current study are available via application from the Mayi Kuwayu Study Data Governance Committee

## Footnotes

1 The Mayi Kuwayu survey notes on this section explain that the ‘Stolen Generations (also known as Stolen Children) are Aboriginal/ Torres Strait Islander people removed from their families by government agencies.
