# Supplementary material for: Exposure to the Family Wellbeing program and associations with empowerment, health, family and cultural wellbeing outcomes for Aboriginal and Torres Strait Islander peoples: a cross-sectional analysis
Source: BMC Public Health. 2023 Aug 18;23:1569. doi: 10.1186/s12889-023-16450-9 (PMC10436403; doi:10.1186/s12889-023-16450-9)
Supplement: Supplementary file 1 — Additional file 1: Appendix 1. Framework for empowerment evaluation outcomes. [file 12889_2023_16450_MOESM1_ESM.docx]

| **Appendix 1. Framework for empowerment evaluation outcomes** | | |  |
| --- | --- | --- | --- |
| **Wallerstein's levels of empowerment** | **Corresponding settings applied in the Tsey & Every evaluation** | **Related empowerment Attributes** | **MK Study Outcome Measures** |
| Personal or psychological empowerment | The Family | Improved perceptions of self-worth and mutability of social environment as evidenced by: empathy and perceived ability to help others; emotional responses to change; critical thinking abilities of root causes of problems, belief in one's ability to exert control; and a sense of coherence about one's place in the world. | *Personal Control* 'How much are you in control of your life?'  *Life Satisfaction* 'How satisfied are you with your life?'  *General Health* 'How would you rate your general health?'  *Family Functionality Scale* |
| Organisational empowerment | The workplace | Stronger social networks and community/ organisation competence to collaborate and solve problems as evidenced by: perceptions of support, satisfaction and community connectedness; and changes in network function and utilisation. | *Cultural Wellbeing scale* |
| Community empowerment | The wider community | Actual improvement in environmental or health conditions as evidenced by: change in public policy; systems level changes; and the community's ability to bring in resources to create healthier environments. | *Decision making* 'In the Aboriginal/ Torres Strait Islander community where I live now local Aboriginal/ Torres Strait Islander people make community decisions.' |
|  |  |  |  |
| Tsey & Every 2000, Adapted from Wallerstein, 1992 |  |  |  |
